# Legitimate Homœopathy

**Published:** 1881-07

**Authors:** C. Pearson

**Affiliations:** Washington, D. C.


### LEGITIMATE HOMŒOPATHY.

C. Pearson, M. D., Washington, D. C.

On the 24th day of November, 1880, I was called to see an old lady, aged ninety-six, who had been left alone by the family, and was found on their return lying at the foot of the stairs in a pool of blood; but being unconscious, she could give no account of how she came there. There was a scalp wound about one inch in length above, and posterior to the left ear, from which blood flowed freely, a small artery having been ruptured; I compressed it, and dressed the sore with a weak solution of arnica, also giving the 20 M. of the same internally, every two hours, till consciousness was returned; this was not rightly established for thirty-six hours. In four or five weeks, the appetite and strength gradually returned; but the sore did not heal; it was covered with a heavy crust, from underneath which a thick yellowish-white matter exuded on pressure.

Some three months after the injury, this crust was removed, revealing a spongy fungus growth on the scalp, about two inches in length by one-half an inch in width, having a red, rough, fleshy appearance, very vascular, *bleeding easily,* and though tender, was not exceedingly sensitive.

For this I prescribed *Phosph.* c. m., three powders in twelve hours, allowing the medicine to act two weeks, by which time there was a slight shrinking of the growth; two more powders of the same medicine were now given, six hours between doses. Improvement quickly seen, resulting in a recovery, April 1st.

Case II.—Master P., aged fifteen, came to our city with hard cough, to which his mother assured me he was subject, but in four or five days, as I had anticipated, he broke out with measles, which ran their usual course; yet his cough continued, becoming more spasmodic, and as whooping began to accompany the paroxysms, its true character could readily be determined. Two weeks after recovering from the measles, in the midst of the whooping-cough, he had chills and fever. The paroxysms being irregular as to time of day, the first coming at 9 P. M. and the next day at 11 A. M.; none came between 10 P. M. and 10 A. M. While for five days there was never twenty-four hours between them, they varied from 11 in the morning to 8 in the evening; no two coming at the same time. The chills were not heavy—scarcely amounting to a shake, and usually lasted about one hour, with fever for two hours, followed by perspiration. There was nothing very marked about the paroxysms, no vomiting, slight thirst, no great amount of pain anywhere, tongue only slightly furred, appetite fair; hence the indications for the remedy were very few. The irregular return of the chills directed me to *Eupat.-purpureum*, three powders of the 50 M. were given, three hours apart, but no perceptible change could be noticed. The only other symptom of importance was in the disposition *to continue covered in bed during the fever*, this suggested *Nux Vom.*, four powders of the 50 M. were given, two hours apart; the chills never returned, though the whooping-cough kept on its course, modified afterwards by *Drosera*.

I have entitled my paper "Legitimate Homœopathy," because every pure coin may be counterfeited, and because I believe these cures were not hybrid, not eclectic, not illegitimate, but veritable cures according to law and science as defined in that bible of homœopathy, the Organon of Sam'l Hahnemann. What have we, as his disciples, to do with shams, with frauds or counterfeits? The world has been filled with these ever since the race began to populate it. Poor suffering humanity has been vomited, purged, bled, blistered and gargled to death for thousands of years; have we nothing better to offer to-day?

Shall we turn the hand on the dial of progress backward, and search for knowledge in the times when men navigated the waters in a dug-out, and cultivated the soil with a stick? When it was believed and taught that "two or three drops of blood, taken from a vein under the tail of a black boar-cat, would cure the epilepsy; and the blood of the ear, would cure the shingles." And is not every step from the Organon of Hahnemann, in the direction of the drug school, a step also in the direction of this same boar-cat? And yet look at the journals flying the homœopathic flag while playing the medical pirate—freebooters, amenable to no law and no system; one drops the name of homœopathic, and becomes the *Medical Times.* Nor need we be surprised at this, as the demand governs the supply. Article IX of the by-laws of the American Institute of Homœopathy, originally made a knowledge of the theory and practice of homœopathy a necessary qualification for membership: this has now been stricken out, though the Institute still retains the name of homœopathy. What inconsistency! If the Institute is to be a kind of go-as-you-please organization, what has homœopathy to do with it, any more than any other pathy? This wedge was entered at Niagara Falls in 1874, a very good place, for "What a fall was there, my countrymen. Then I, and you, and all of us fell down, while bloody treason flourished over us." This was done because, forsooth, four years previously Carroll Dunham had advocated freedom in medical thought and action. A certain amount of freedom is well enough; but to acknowledge no law and no principles becomes the worst kind of anarchy, and, if tolerated, will destroy any government, whether political or medical. Already we see members, who aspire to the presidency, repudiating even the name of homœopathy. One says the great mistake of his life was in accepting a diploma from a homœopathic college; and, we might add, it was a great mistake of the college as well. Another allows his name to be used to encourage the sale of a patent medicine, and boasts of being only a physician—that if the old school have found any good thing, he is at liberty to use it, without any regard to homœopathy. Another still publishes a book, in which he writes himself down an ass, in a vain attempt to show that the schools both use the same drugs, and that, therefore, there is not much difference between them. Across the water, the picture is equally gloomy. The disgraceful treatment of Lord Beaconsfield by Dr. Kidd caused the British Congress to repudiate him—not that they were less crude than he; but because he had the candor to admit that he was not an homœopathist; that he had rejected pretty much everything of Hahnemann's teaching, except the law of the similars; and as this was no discovery of Hahnemann's, having been admitted a thousand years before he was born, of course, with the law alone there could be no claim to homœopathy—this came from Hahnemann, the law from Hipocrates. I prefer Hahnemann sober, to Hahnemann drunk, says Dr. Kidd; or, to make his meaning still plainer, he might have said, Hahnemann as an allopath, to Hahnemann as a homœopath. And the latter, were he living, would probably prefer Kidd sane, to Kidd crazy.

It is remarkable how these men grasp at anything Hahnemann said or did in his early investigations, and how they prefer to grope in the twilight, rather than to follow him into the strong reflection of the noon-day sun. But he wrote his Organon, thank God! and there, like a flaming sword, it stands in their way; and there, notwithstanding all their cunning devices, their gross misrepresentations and blank falsehoods, it will continue to stand when

> " Moths deform in shapeless tatters
> Their unknown pages."

Now what claim have such men to homœopathy? Hahnemann called them the "new mongrel sect," and exclaims, "who would honor such a light-minded and pernicious sect by calling them after the difficult, yet beneficent art of homœopathic physicians!" He says, "It is in this way that a madman who has forced his way into the workshop of an artist seizes, with open hands, upon all the tools within his reach for the purpose of finishing a work which he finds in a state of preparation. Who can doubt but that he will spoil it by the ridiculous manner in which he goes to work, or perhaps even destroy it entirely."

And yet these "madmen" have the audacity now to tell us that Hahnemann himself, the master workman, spoiled it by his eccentricities, his small doses, his dynamic and psoric theories. "Oh, wise judge! This master mind declares it is as impossible that there should be any other true method of curing dynamic diseases (that is, those not surgical) besides homœopathy, as that more than one straight line can be described between two given points."

Again, in speaking of this class of physicians, he says: "They cannot accomplish that which the true homœopathist is capable of doing, and yet they falsely declare themselves my disciples." He lays it down as a truth—which is the invaluable property of pure homœopathy, "that the best dose of medicine is ever the smallest, that the practice of the new mongrel sect, consisting in a combination of allopathy and homœopathy, will separate them by an immeasurable gulf from homœopathy." If this gulf of late years has been widening, it is no fault of ours; we fight under the old banner. If, in exercising their liberty of "medical opinion and action," others have left us, it is their loss, not ours. Years of wandering towards the pyramids of the dead past, will serve to show them that far in their rear and in the van of progress, they have left the temple of pure homœopathy, radiant in the sunlight of eternal truth.

To-day (May 25th) as I was writing this, I was called in haste to see a lady at the climacteric period of life, the mother of a large family, found her frantic with gastralgia with which for years she had often been troubled. A number of persons were in the sick-room, all busily engaged heating irons, ironing paper, etc., to be applied to her stomach. All was excitement; each one had something to do; the same lively scene was presented that I had often witnessed in my erratic days, when I too worked with hot fomentations and drop doses of the first or third dilution, till the perspiration streamed from my face. I thought of the sleepless nights and anxious days I had spent with just such cases, in the vain endeavor to hit upon something that would induce the pain to leave; and yet it kept on all the same. This patient called loudly for chloral, said she could not live without it; that her physician had found that nothing else would do any good; that she had then been having the pain for eight hours, and could endure it no longer. She described the pain as being hard, cramping, *burning* from the cardiac orifice of the stomach through to the back; not much thirst, no fever, slight nausea, but no vomiting. She was very *restless*, constantly changing position, walking the floor or rolling on the bed and screaming with pain. She asked again and again, if I would not give her Chloral. I answered very decidedly that I would not. "I believe," said a lady present, who seemed to have borrowed her argument from the eclectics, "in giving anything that will relieve pain the quickest." I replied that I did, too, and that the proper homœopathic remedy was that very thing. I therefore prepared the 50 M. of *Arsenicum* in water, gave a dessert-spoonful and held my watch to note results. In five minutes, the out-cry and restlessness were less; in eight minutes, the inspirations were slower and fuller; in ten minutes, she said she felt easier; and in twelve minutes, the pain was entirely gone and did not return. Now what becomes of the argument that homœopathic remedies are slow to act? Could chloral or any other narcotic, by benumbing the senses, have smothered the sensation of pain in less time than it was *cured* in this instance. And yet the eclectics tell us there is nothing in the high or fluxion potencies; that all traces of the drug had left long ago, or that the pain was about to leave, at any rate. How strange that it is usually about to go by the time the proper homœopathic remedy and potency are applied; and yet it will as stubbornly resist the eclectic's art, as he does the only law to which it yields. "Ye Gods, it doth amaze me," to hear the frivolous excuses made for being ignorant; but the work of the iconoclast is not finished; the idols in the temple of Baal must be broken, though their votaries oppose every advance of truth.

> There must be strife if error dies,
> Or truth would ever triumph o'er it;
> The ages hence award a prize,
> To pioneers who bravely bore it.

---

The Committee on Publication respectfully report the above papers, as presented to the Association, to THE HOMŒOPATHIC PHYSICIAN for publication.

AD. FELLGER,
GEO. H. CLARK,
E. J. LEE,
*Committee.*

